# Base Oils and Formulated Transmission Oils for Electrical Vehicles: Thermophysical and Tribological Properties

**DOI:** 10.3390/ma18061207

**Published:** 2025-03-08

**Authors:** José M. Liñeira del Río, Alonso Alba, Martín Gómez Martínez, Alfredo Amigo, Josefa Fernández

**Affiliations:** 1 Laboratory of Thermophysical and Tribological Properties, Nafomat Group, Department of Applied Physics, Faculty of Physics, Institute of Materials (iMATUS), Universidade de Santiago de Compostela, 15782 Santiago de Compostela, Spain; alonso.alba@rai.usc.es (A.A.); martin.gomez.martinez@rai.usc.es (M.G.M.); josefa.fernandez@usc.es (J.F.); 2 Laboratory of Thermophysical and Surface Properties of Liquids, Department of Applied Physics, Faculty of Physics, University of Santiago de Compostela, 15782 Santiago de Compostela, Spain; alfredo.amigo@usc.es

**Keywords:** lubricants, surface tension, contact angle, tribology

## Abstract

The aim of this research is to analyze the thermophysical, wettability, and tribological properties of some base oils of different nature (synthetic and mineral), as well as of formulated oils, to find potential transmission oils for electrical vehicles. Regarding the thermophysical properties, viscosity, density, and viscosity index were analyzed. Surface tension and contact angle were also measured to obtain the wettability performance of tested lubricants. The highest viscosities were found for the PAO8 oil and the lowest for the G-III 3 base oil, while the highest densities were found for the formulated oils. Concerning wettability performance, the surface tensions of PAOs and G-IIIs rise gradually with an increase in viscosity, the surface tension being the greatest for G-III 6 and the lowest for G-III 3. Finally, in the tribological characterization, the lowest coefficients of friction and produced wear were found with the formulated lubricants, due to the presence of an additive package.

## 1. Introduction

Today, the emergence of electric vehicles (EVs) as an alternative to vehicles with internal combustion engines (ICEs) has appeared in our society, with the main objective of minimizing fuel use and decreasing greenhouse gas emissions [1,2,3]. An interesting way to optimize the performance of these EVs is to tribologically improve their mechanical components, thereby reducing the energy loss in wear and friction. For this aim, the use of appropriate lubricants that result in minimal tribological losses is of special importance. In certain EVs, the components of the electric motor and transmission are enclosed within a single cover. In such circumstances, the electric transmission fluids (ETFs) must possess the following characteristics: low viscosity, inhibited copper corrosion, compatibility with polymers, and suitable electrical properties [4,5,6,7]. The utilization of low-viscosity lubricants is imperative, given the elevated operational speeds and torque of the mechanical components in EVs. A decrease in oil viscosity leads to a reduction in viscous drag and viscous heating, as well as an enhancement in heat transfer [8,9]. However, a decrease in lubricant viscosity may result in a transition from full film to boundary lubrication. This transition is likely to result in significant surface contact and consequent substantial wear. To avoid these issues, several antifriction or antiwear additives should be used [10,11]. These base oils are generally of different natures: mineral, synthetic, or vegetable. Therefore, it is interesting to have a tribological data collection of low-viscosity lubricant oils of different natures in order to know what type of oils could be developed to design potential transmission fluids for EVs. Another salient property of transmission fluids is their capability to spread on metal surfaces, thereby influencing their wettability. Wettability is typically characterized by measuring the contact angle and/or surface tension. The contact angle, θ, is the angle formed between the surface of an oil and a solid surface upon contact. The value of the angle is primarily determined by the interplay between the adhesive forces of the oil and the solid, as well as the cohesive forces inherent to the liquid itself. It is imperative to examine the dependence of these properties on temperature. Studies on these properties have been reported for several fluids like ionic liquids [12] or lubricants [13,14,15]. Hence, it is useful to know how the contact angle will behave at the operating temperature of EV transmissions, indicating whether there is an enhanced wettability and whether it could lead to the formation of a tribofilm that might impede surface contact and thus improve the antifriction and antiwear behavior of a lubricating oil.

In this regard, it is also interesting to know how additives (as antifriction or antiwear) could modify the viscosity of oils, because a substantial increase in this property could result in a shift in the operational range of the lubricant, thereby compromising its efficacy as a transmission fluid. Therefore, the goal of this research is to study eight different low-viscosity oils with different natures through thermophysical properties, wettability, and tribological performance to achieve potential transmission fluids for EVs. Thus, this group of eight oils is composed of two polyalphaolefin (PAO) synthetic oils (PAO6 and PAO8), three group III (G-III) mineral oils (G-III 3, G-III 4 and G-III 6), and three ATF (automatic transmission fluid) formulated lubricants (ATF CVT, ATF DTC, ATF VI). To our knowledge, there is no research that has studied the tribological and wettability properties of different base oils. The authors consider that these studies are necessary to design potential formulated nanolubricants based on these oils, since it has recently been proven that the combination of nanoparticles (NPs) in existing lubricants can result in a significant decrease in friction and wear for mechanical components [16,17,18,19,20].

## 2. Materials and Methods

### 2.1. Materials

The polyalphaolefin 6 base oil, PAO6, polyalphaolefin 8 base oil, PAO8, ATF CVT, ATF DTC, ATF VI, G-III 3, G-III 4, and G-III 6 were provided by REPSOL (Madrid, Spain). All of them are low-viscosity oils. PAO6 and PAO8 are synthetic base oils, and ATF CVT, ATF DTC, and ATF VI are paraffinic and additivated oils, whereas G-III 3, G-III 4, and G-III 6 are paraffinic base oils. The numbers 3, 4, 6, and 8 in the name of the lubricant base oils indicate the kinematic viscosities in cSt at 100 °C. Table 1 displays the main physical properties of the oils tested in this research.

### 2.2. Refractive Index of Oil Samples

The refractive index for the eight oils was measured using a Mettler Toledo RA-510M refractometer (Mettler-Toledo, LLC., Columbus, OH, USA), which can operate at temperatures between 288.15 K and 313.15 K. The refractometer cell consists of an inverted conical stainless steel cavity with a polished sapphire prism at the base of the cone. Approximately 0.25 mL of sample is required to determine the refractive index. Specifically, in this research, the refractive index of the lubricant oils was determined at 298.15 K and 313.15 K.

### 2.3. Density, Viscosity, and Viscosity Index of Lubricant Oils

A Stabinger Anton Paar SVM 3000 viscometer was used to analyze the density and viscosity of the different oils. With this instrument, both properties can be determined at atmospheric pressure in the temperature range from 233.15 K to 378.15 K and in a viscosity range from 0.2 mPa·s to 20 Pa·s. The values of the density and dynamic and kinematic viscosity of oils between temperatures of 278.15 K and 373.15 K in 5 K intervals were obtained. Moreover, the viscosity index (VI) was also determined with the same instrument according to the ISO2909 [21] and ASTM D2270 [22] standards, with an estimated uncertainty of 2.7 [23]. The temperature in the cell is monitored via an integrated thermostat with a Peltier and is measured with a Pt100 thermometer with an expanded uncertainty (k = 2) of 0.02 K from 288.15 to 378.15 K. Approximately 3.5 mL of each oil sample was used for these measurements. Oils are introduced through the upper cavity of the U-tube cell of the viscometer, simultaneously filling the two measuring cells: one for density and the other for viscosity. Therefore, the two properties are measured at the same time. More information concerning this experimental instrument can be found in prior research [24].

### 2.4. Contact Angle Analysis and Surface Tension

Surface tension measurements were performed using a Drop Volume Tensiometer (Lauda TVT, Marlton, NJ, USA), which determines the surface tension by means of the volume of a falling drop according to Tate’s law [25]. Each oil is placed into a syringe of 2 mL and ejected through a 1.37 mm inner radius needle. It should be noted that the temperature of each oil in the syringe is monitored by a thermostatic bath to maintain it at the desired temperature (in this case, 298.15 or 313.15 K). The uncertainty of the temperature is u(T)/K = 0.03. For each individual test, around 30 drops are used to obtain an average value. The uncertainty of the surface tensions varies with the oil ranging from 0.07 to 0.12 mN m^−1^.

For contact angle measurements, a Phoenix MT-A tester was used to obtain the contact angle of the lubricants on AISI 420 stainless steel plates. The calibration of the equipment camera is performed using a glass plate provided by the manufacturer. This plate is equipped with reference graduation marks, which are designed to align with various contact angles. Each lubricant oil is located into a syringe that, with the assistance of the instruments’ motor, produces a drop of oil which falls on the steel plate. This plate is placed on a thermostated surface with a bath to hold the required temperature (in this case, 298.15 or 313.15 K) during the measurement time. The uncertainty of temperature is u(T)/K = 0.5. The result of the measurement is the evolution of the contact angle over time, exactly its value every second for 60 s. For each lubricating oil, three series of tests were carried out, from which the average of all the values was calculated. The estimated uncertainty in the contact angle tests was 2%. More information regarding this apparatus is reported in a previous article [26].

### 2.5. Tests of Friction and Wear Examination

Friction tests with all the base oils and formulated lubricants were performed using a T-PTD200 tribology cell connected to an Anton Paar MCR 302 rheometer (Anton Paar GmbH, Graz, Austria). The configuration consists of the disposition of a rotational ball-on-three-pins. The ball is placed at the end of a vertical tube that is controlled by the motor of the rheometer, while the pins located at the base of a container are sited at an angle of 45° relative to the tube. During the tests, the ball, which provides support for a vertical axial force applied by the rheometer, rotates around on the pins. More characteristics regarding the disposition as well as the different settings of the friction tests are reported in Table 2 and in previous articles [27,28]. Three replicates for each oil were carried out to determine the average friction coefficient. Lastly, the wear generated in each pin was determined using a 3D optical profilometer (Sensofar S Neox, Sensofar, Barcelona, Spain) with a confocal mode and a 10× objective. The wear characteristics measured in the three pins were wear scar diameter (WSD), wear track depth (WTD), and worn area. It should be noted that 3 replicates of wear measurements were performed for each lubricant to ensure good reproducibility. Furthermore, the profilometer specifications are briefly summarized in Table 3. This instrument was calibrated according to the ISO 25178 standard [29].

## 3. Results

### 3.1. Refractive Index Results

As mentioned previously, in this research, the refractive index of the studied lubricant oils was measured at 298.15 K and 313.15 K. Table 4 indicates the obtained values for all the tested lubricants, showing that the formulated lubricants (ATF CVT, ATF VI, and ATF DCT) have the highest refractive index. This fact may be due to the additive package. Furthermore, it can also be seen that for each base oil family (mineral or synthetic), the refractive index increases as viscosity rises; thus, for example, the PAO8 refractive index is higher than that of PAO6.

### 3.2. Density, Viscosity, and Viscosity Index Results

**Figure 1:**
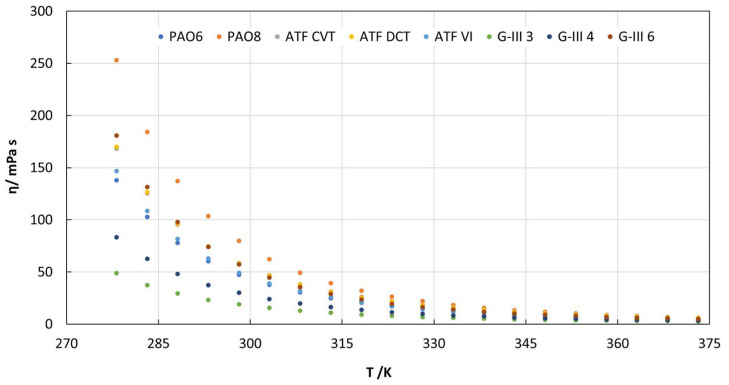
Dynamic viscosities found for all the tested lubricant oils.

As mentioned above, the densities and viscosities of the base and formulated lubricants were measured at temperatures ranging from 278.15 K to 373.15 K. Thus, as can be observed in Figure 1 and Table S1, at 278.15 K, the dynamic viscosities of the tested oils ranges between 253 and 49 mPa s, for PAO8 and G-III 3, respectively. At 313.15 K, the viscosities for all the tested lubricants are very similar (around 5 mPa s). By observing the viscosity results obtained, the trends of the lubricants can be established from more to less viscous as follows: PAO8, G-III 6, ATF DCT, ATF CVT, ATF VI, PAO6, G-III 4, and G-III 3. Therefore, there is not a clear trend between the family of different lubricants as occurred with the refractive index. The trend agrees with the viscosity grade of the lubricants.

**Figure 2:**
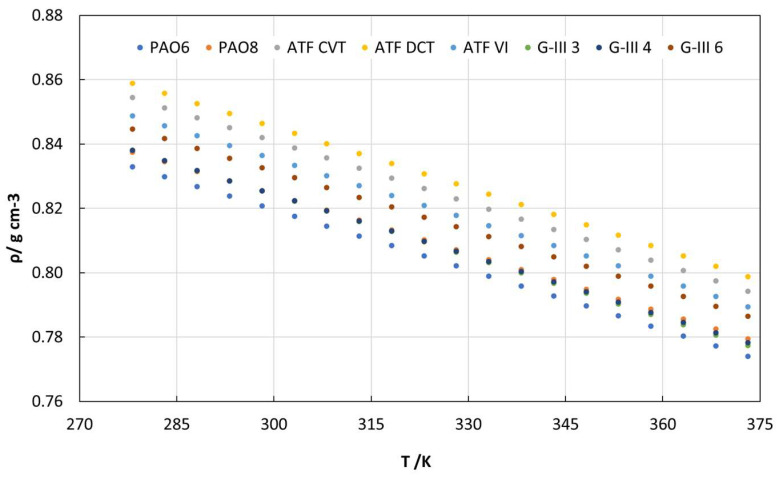
Density values achieved for all the tested lubricant oils.

Regarding the densities of the eight lubricant oils, Figure 2 and Table S2 show that the highest densities were obtained for the formulated oils (ATF DCT, ATF CVT, and ATF VI). This fact may be because these formulated lubricants have an additive package that can increase the density of the oils. On the other hand, it is also observed that the PAOs, especially PAO6, have the lowest densities, given that PAO6 has the lowest molecular mass among the PAOs, being a salient subject for consideration.

**Figure 3:**
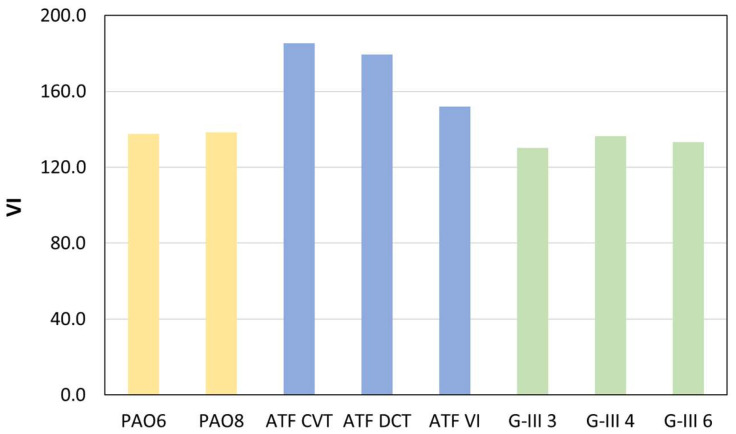
Viscosity index values obtained for all the studied lubricant oils.

Regarding the viscosity index (VI) measurements, Figure 3 displays the values found for the base and formulated oils. The results obtained are quite interesting because the formulated oils have the highest VI values, whereas the PAOs are the base oils with the highest VI, and the G-III base oils are the oils with the lowest VI. Therefore, it seems that the formulated oils (containing mineral base oils as G-III oils) have different additives to improve their performance with temperature variation, such as, for example, VI improver additives.

### 3.3. Contact Angle and Surface Tension and Results

**Figure 4:**
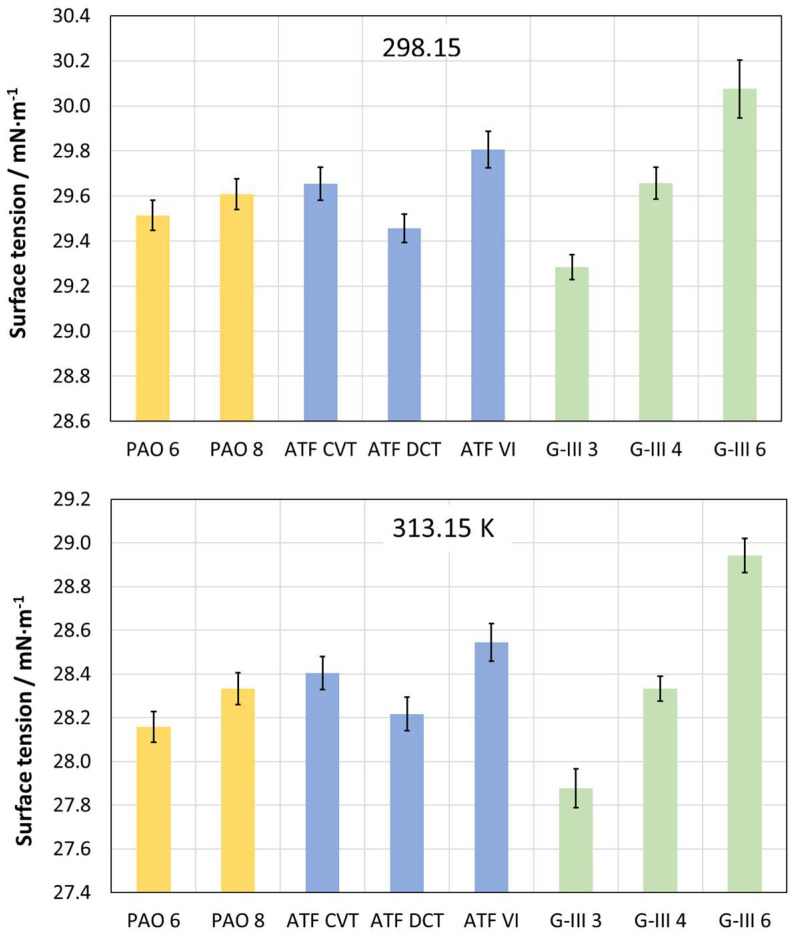
Surface tension values obtained for the different tested oils at 298.15 and 313.15 K.

Figure 4 and Table 5 depict the experimental values of the surface tension, γ, of the formulated and base oils for temperatures of 298.15 K and 313.15 K. Figure 4 clearly indicates that the surface tensions of PAOs and G-III oils increase with a rise in viscosity. It can also be seen that the oil with the highest surface tension for both tested temperatures is the G-III 6, and the one with the lowest is the G-III 3. Similar results were also observed by Marques et al. [26], who studied the surface tension of different PAO base oils (PAO6, PAO20, PAO32, and PAO40); they also observed that as the viscosity of PAO increases, the surface tension rises.

**Figure 5:**
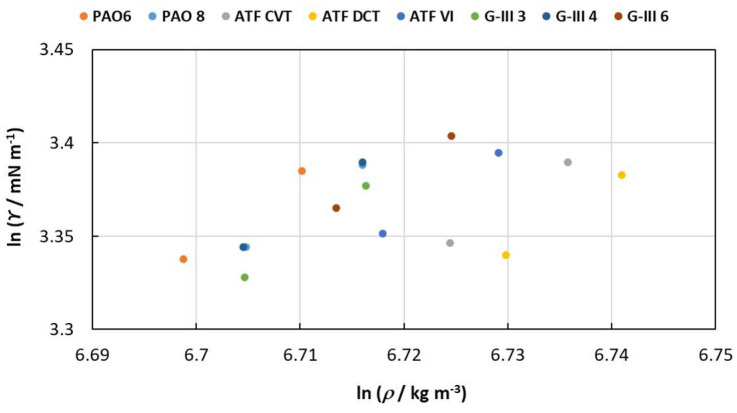
Linear relationship between ln (γ) and ln (ρ) for the studied lubricant oils.

Through the relationship planned by Pinheiro et al. [17], the linear dependency relating to ln (γ) and ln (ρ) was studied:

(1) $$\ln(\gamma /mN\cdot m^{-1})=C+\lambda \;ln\;\left(\rho /kg\cdot m^{-3}\right)$$

in which the parameters C and λ are the coefficients of the linear regression representation. Figure 5 illustrates that the linear performance is consistent across all oils, showing a similar slope for all the lubricants studied. A clear distinction can be observed between two classes: base oils (left part of the Figure 5) and formulated oils (right part).

**Figure 6:**
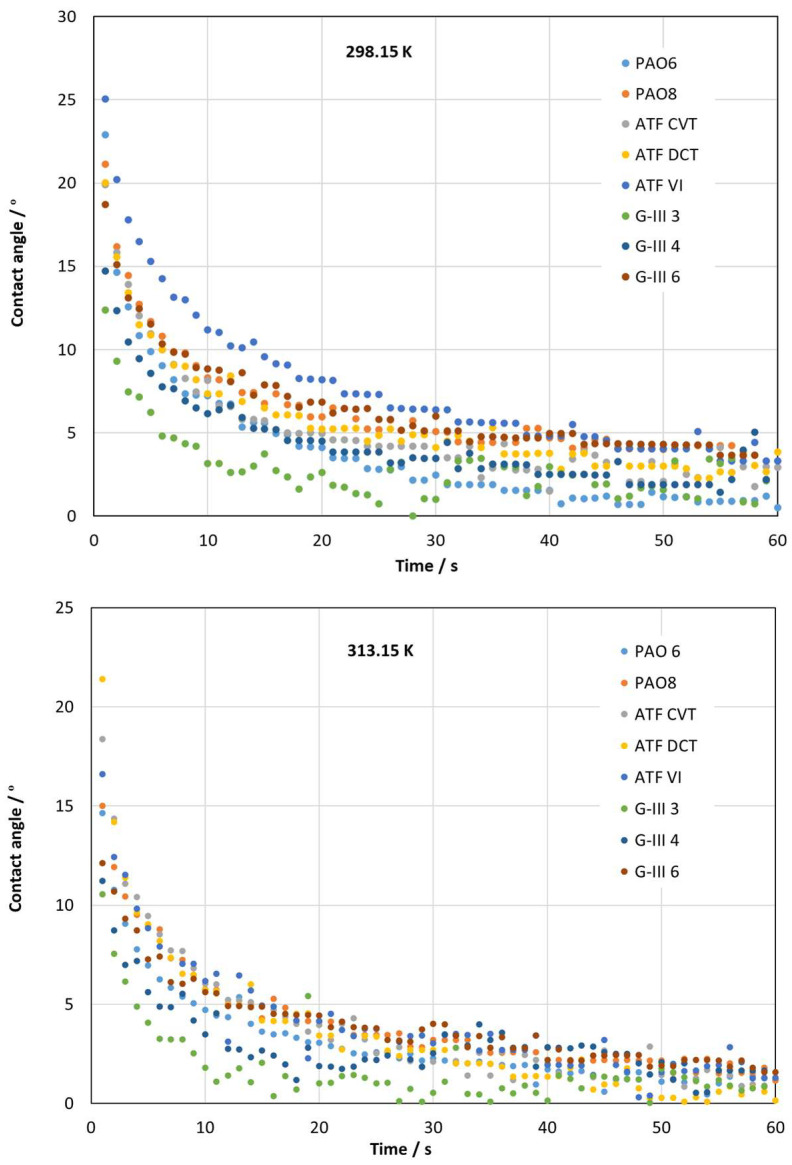
Contact angle values achieved for the tested lubricants at 298.15 and 313.15 K.

The measurements of contact angle for the different formulated and base oils are shown in Figure 6 and Table 6. At the low temperature (298.15 K), it is observed how the PAO6 base oil presents the highest contact angle values and G-III 3 the lowest. On the other hand, at the high temperature (313.15 K), the contact angle values are similar for all the tested lubricants, but also being lowest for the G-III 3. It is worth noting that the observed variations can be attributed to the combined uncertainty of measurements. Additionally, it was detected that as the temperature increases, the contact angle values decreases. Consequently, at the operational temperature of EV transmissions (approximately 393 K), the contact angle will be reduced, indicating an enhanced wettability and the formation of a tribofilm that might impede surface contact [31]. This contact angle–temperature dependence was also observed by Marques et al. [26] who studied the contact angle of different PAOs, finding that when the temperature is increased the contact angle values drop. Additionally, they also found that for high viscosity PAOs, the contact angle is greater than those observed for low-viscosity PAOs. In our case, this trend is also reached, since contact angle values for PAO6 are greater than those obtained for PAO8. Furthermore, Tetteh et al. [32] studied the wettability of surfactants on calcite, observing a clear correlation between the number of ethoxy groups and the wettability efficiency of surfactants, whereby the higher number of ethoxy groups results in a greater degree of wettability.

**Figure 7:**
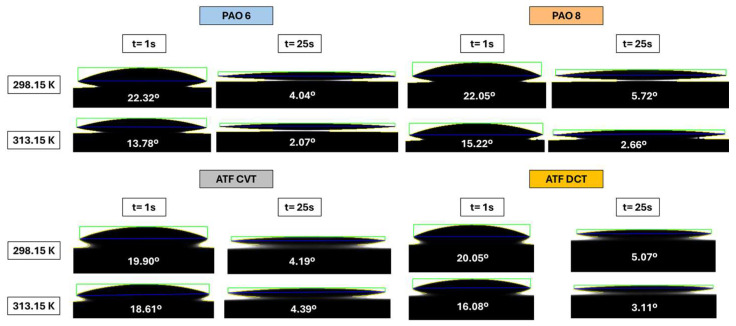

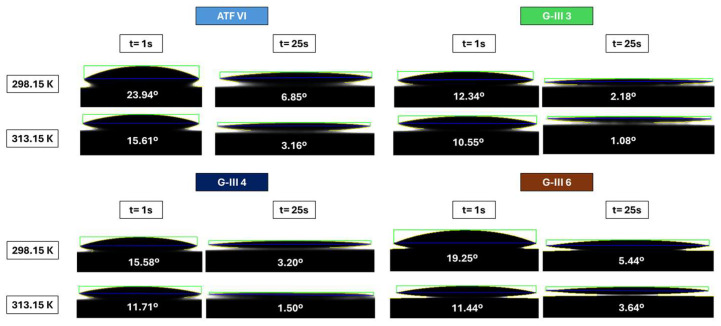
Contact angle photographs for all the studied lubricants at t = 1 s and t = 25 s for the temperatures 298.15 and 313.15 K.

To better visualize the different contact angles measured for all base oils and formulated oils, Figure 7 shows the contact angles at t = 1 s and t = 25 s for all the studied lubricants at the two operating temperatures, 298.15 K and 313.15 K.

Contact angle is linked with surface tension by Young’s equation at the different interfaces [33]:

(2) $$\cos\left(\theta \right)=\frac{\gamma_{SM}-\gamma_{SL}}{\gamma_{LM}}$$

in which γ_SM_ corresponds to the surface tension between the surface and surrounding environment, γ_SL_ is between the surface and liquid, and γ_LM_ is between the liquid and nearby environment. Consequently, when the γ_SL_ grows, the cos (θ) decreases, increasing the liquid contact angle. Hence, the results found for the surface tension and contact angles for all the studied lubricants are consistent. Thus, for example, for polyalphaolefins, PAO6 has lower surface tensions and contact angles in comparison to those of the PAO8 oil.

Additionally, the influence of wettability on lubrication is normally depicted by the spreading parameter, S, that is calculated as follows:

(3) $$S=\gamma_{SM}-{(\gamma }_{SL}-\gamma_{LM})$$

Combining Equations (2) and (3), the spreading parameter can be identified as the variation among the adhesion between the liquid and surface (W_A_) and the cohesion of the liquid (W_C_):

(4) $$S=\gamma_{LM\;}cos\;\theta -\gamma_{LM}=\gamma_{LM}\;\left(cos\;\theta -1\right)$$

Thus, the adhesive and cohesive forces can be calculated:

(5) $$W_{A}=\gamma_{LM\;}cos\;\theta$$

(6) $$W_{C}={2\;\gamma }_{LM\;}$$

Therefore, the spreading parameter can be explained nearly as:

(7) $$S=W_{A}-W_{c\;}{\approx \gamma }_{LM\;}\left(cos\;\theta +1\right)-2\;\gamma_{LM}$$

Therefore, if S > 0, W_A_ > W_C_, and the oil spreads totally on the surface to reduce its surface energy. On the other hand, if W_C_ > W_A_, the oil creates a drop on the surface owing to cohesive forces among the liquid molecules that are connected more effectively to others. The values of the spreading parameter obtained from Equation (7) with the experimental values of contact angle and surface tension for all the lubricant oils are presented in Table 7. It can be seen that the spreading parameters, S, have negative values for all the tested lubricants, which means that the cohesive forces of lubricant oils are superior to the adhesion forces at the liquid–solid interface, and the drop forms a sphere with a persistent contact angle due to the increased cohesion forces between liquid molecules. Similar spreading parameter values were achieved by Chandra Behera et al. [34] for different metal-working fluids and lubricating Inconel 718 alloys, obtaining for all combinations negative spreading parameter values ranging from −10 to −75.

### 3.4. Friction and Wear Results

**Figure 8:**
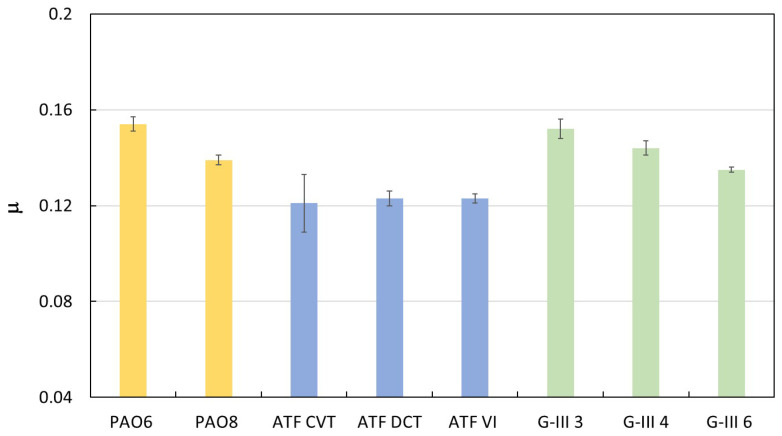
Average coefficients of friction (μ) attained with the different lubricant oils at 393.15 K.

Friction pure sliding tests were performed for all the different base and formulated oils: PAO6, PAO8, ATF CVT, ATF DTC, ATF VI, G-III 3, G-III 4, and G-III 6. The average values of the coefficient of friction, μ, are shown (Figure 8 and Table 8) for all the lubricants. As expected, the friction coefficients achieved with the formulated oils (ATF CVT, ATF DTC, ATF VI) are lower than those of the PAOs and G-III base oils. In Figure 8, it can be seen that for PAO and G-III oils, the friction coefficient decreases as the viscosity increases. Thus, the friction coefficient of PAO8 is lower than that of PAO6, and the friction coefficient of G-III 6 is lower than those of G-III 4 and G-III 3. Specifically, for all the lubricants tested, the maximum coefficient of friction was achieved for PAO6 with a value of 0.154, and the lowest was for ATF CVT with a value of 0.121.

**Figure 9:**
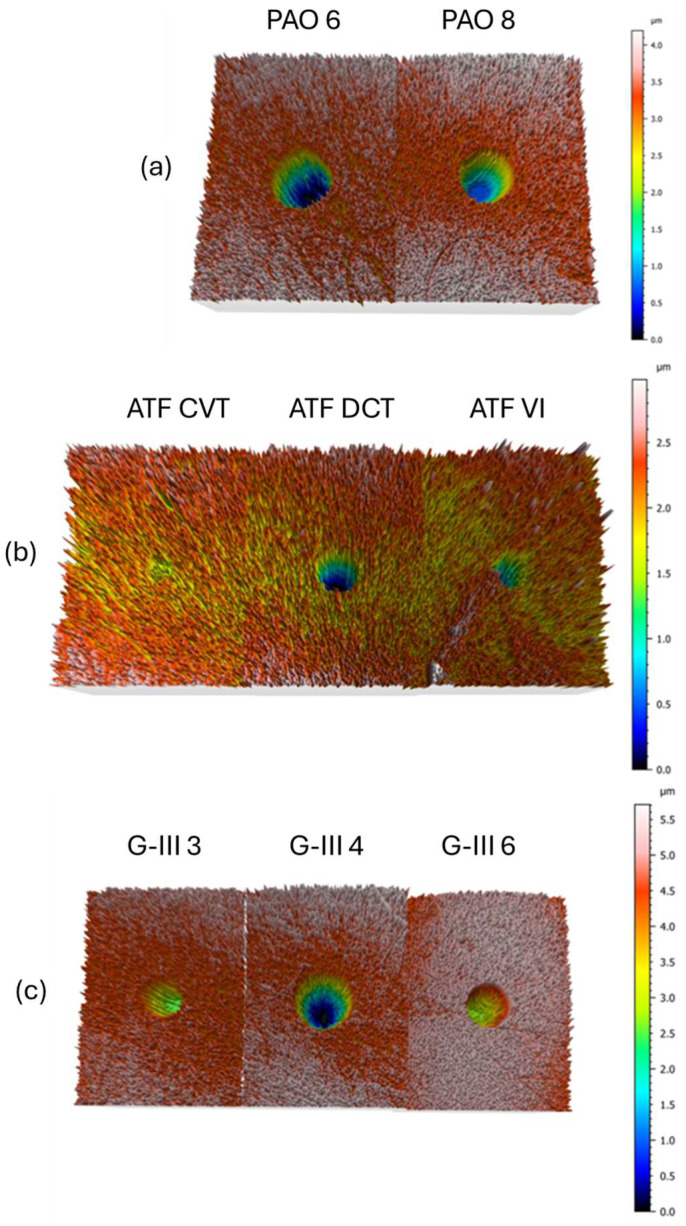
Wear 3D profiles found for the steel pins tested with lubricant bases, PAOs (**a**), formulated oils (**b**), and GIII oils (**c**).

For the wear generated in the pins after the tribological tests, the 3D profile measurements (Figure 9) display that the worn surfaces lubricated with formulated oils (ATF CVT, ATF DTC, ATF VI) have lower wear in comparison to those with base oils. These results were expected, since these lubricants present an additive package. Regarding the base oils, it is observed that the wear produced in pins lubricated with PAOs is bigger than that produced in the pins lubricated with G-III oils. In particular, the oil with the worst tribological performance is PAO6 with a WSD of 435 μm, a WTD of 2.60 μm, and a worn area of 806 μm^2^. On the other hand, the oil with the best wear results is the ATF CVT with a WSD of 435 μm, a WTD of 2.60 μm, and a worn area of 806 μm^2^. Therefore, the tribological results are very interesting to know if a studied lubricant can be improved easily or not. For instance, in previous research [37], wear improvements of ATF VI were reached by the addition of Al_2_O_3_ nanoparticles, having the best wear reduction with the ATF + 0.10 wt% Al_2_O_3_ nanolubricant, with a reduction of 45% in the diameter of the wear scar. Based on this previous result and the present research, it seems that, for example, for formulated ATF DCT, the wear reductions could even be more important, since the wear produced with this lubricant is greater than that achieved with ATF VI. Thus, with the data of this research, it would be easier to develop improved potential transmission lubricants for electric vehicles.

## 4. Conclusions

In this investigation, the following findings were obtained:

- Refractive index values were higher for the formulated oils than for base oils. For G-III and PAO base oils, the refractive index increased as the viscosity rose.
- The dynamic viscosities of the tested oils at 278.15 K ranged between 253 and 49 mPa s, for PAO8 and G-III 3, respectively. On the other hand, at 373.15 K, all the lubricants had similar viscosities (between 3 and 8 mPa s).
- The densities of the formulated lubricants (ATF DCT, ATF CVT, and ATF VI) were the highest, whereas PAOs had the lowest densities (PAO6).
- The surface tensions of PAOs and G-IIIs rose gradually with an increase in viscosity, the surface tension being greatest for G-III 6 and lowest for G-III 3.
- As the temperature increased, the contact angle values decreased. Therefore, at the operational temperature of EV transmissions, contact angles will be low, indicating increased wettability and the formation of a tribofilm that could prevent surface contact.
- As expected, the lowest friction coefficients were obtained with the formulated lubricants, due to the presence of the additive package. The highest friction coefficients were obtained for the low-viscosity PAOs and G-IIIs, PAO6 and G-III 3, respectively.
- The lowest wear generated in the pins was obtained for those lubricated with formulated oils. Regarding the base oils, the tested pins lubricated with PAOs presented greater wear than those lubricated with G-IIIs.
- Based on the results, it should be interesting to study the tribological performance of these base oils with different nanoadditives to design potential EV lubricants and compare them with the tribological performance obtained by the formulated oils.
- Before using these lubricants in EVs, more studies are needed, such as tribological tests with long times at high loads, to estimate their possible degradation with time.

## Figures and Tables

**Table 1:** Main physical properties of the tested oils.

Lubricant Oil	Nature	Density at 313.15 K g cm^−3^	Dynamic Viscosity at 313.15 K mPa s
PAO6	Synthetic	0.8114	24.853
PAO8	Synthetic	0.8163	39.470
ATF CVT	Mineral	0.8325	31.411
ATF DCT	Mineral	0.8370	30.943
ATF VI	Mineral	0.8271	25.814
G-III 3	Mineral	0.8159	10.889
G-III 4	Mineral	0.8162	16.298
G-III 6	Mineral	0.8234	28.850

**Table 2:** Main characteristics of the tribological pairs and friction test settings.

Tribology Cell T-PTD200		
Ball-on-Three-Pins Disposition		Peltier HPTD200
Pairs	Test Information	
100Cr6 steel ball: 12.7 mm diameter and 0.15 µm Ra 100Cr6 pins: 6 mm diameter and a 0.3 µm Ra Specimens: Hardness: 58–65 HRC Young’s modulus: 190–210 GPa Poisson ratio: 0.29	Sample: 1.1 mL Tribological force (F_N_): 9.4 N Maximum contact pressure: 1.1 GPa Distance: 340 m Speed: 213 rpm	120 °C

**Table 3:** 3D profilometer specifications and determined wear parameters.

Sensofar S Neox	
Specifications	Measured Parameters
Analysis mode: confocal Objective: 10×	Wear scar diameter (WSD) Wear track depth (WTD) Worn area (Area) Surface roughness (Ra): ISO 21920-2:2021 standard [30] Gaussian filter: 0.08 mm

**Table 4:** Refractive index values for all the studied lubricant oils.

	G-III 6	G-III 4	G-III 3	ATF CVT	ATF VI	ATF DCT	PAO6	PAO8
298.15 K	1.46223	1.46210	1.46122	1.46643	1.46564	1.46612	1.46012	1.46231
313.15 K	1.45668	1.45642	1.45546	1.46084	1.46145	1.46045	1.45761	1.45814

**Table 5:** Surface tension values, γ, and its uncertainties, σ, of the tested oils.

	298.15 K		313.15 K	
	γ/mN m^−1^	σ/mN m^−1^	γ/mN m^−1^	σ/mN m^−1^
PAO6	29.51	0.07	28.16	0.07
PAO8	29.61	0.07	28.33	0.07
ATF CVT	29.65	0.07	28.40	0.08
ATF DCT	29.46	0.06	28.22	0.08
ATF VI	29.81	0.08	28.55	0.09
G-III 3	29.29	0.06	27.88	0.09
G-III 4	29.66	0.07	28.33	0.06
G-III 6	30.07	0.12	28.94	0.08

**Table 6:** Mean contact angle values (θ) at t = 10 s and their standard deviations (σ) for the tested lubricants.

	298.15 K		313.15 K	
	θ/°	σ	θ/°	σ
PAO6	7.1	0.4	4.7	1.0
PAO8	9.2	1.7	6.0	0.2
ATF CVT	9.7	2.8	6.8	0.9
ATF DCT	8.1	0.8	6.0	0.5
ATF VI	12.0	2.3	5.7	0.6
G-III 3	3.8	0.8	3.0	1.0
G-III 4	5.9	0.3	3.9	0.7
G-III 6	8.7	0.5	5.9	0.8

**Table 7:** Spreading parameter values (S) for the tested lubricants at 298.15 and 313.15 K.

	PAO6	PAO8	ATF CVT	ATF DCT	ATF VI	G-III 3	G-III 4	G-III 6
298.15 K	−65.35	−79.52	−66.40	−86.90	−32.67	−54.72	−35.50	−88.96
313.15 K	−35.85	−36.07	−35.42	−35.19	−31.05	−73.72	−33.91	−35.88

**Table 8:** Mean coefficients of friction, μ, mean wear scar diameter, WSD, mean wear track depth, WTD, and mean worn area for all the lubricants oils at 393.15 K.

Lubricant	µ	σ	WSD/μm	σ/μm	WTD/μm	σ/μm	Area/μm^2^	σ/μm^2^
PAO6 [35]	0.154	0.003	435	10	2.60	0.19	806	65
PAO8 [36]	0.139	0.002	392	14	2.35	0.17	605	52
ATFCVT	0.121	0.012	236	11	0.55	0.08	66	10
ATFDCT	0.123	0.003	331	36	1.38	0.21	294	54
ATFVI [37]	0.123	0.002	289	16	0.82	0.09	163	27
G-III 3	0.152	0.004	347	31	1.53	0.33	358	94
G-III 4	0.144	0.003	381	46	2.05	0.41	589	62
G-III 6 [38]	0.135	0.001	366	18	2.11	0.19	607	44

## Data Availability

The original contributions presented in this study are included in the article/Supplementary Material. Further inquiries can be directed to the corresponding author.

## Supplementary Materials

The following supporting information can be downloaded at: https://www.mdpi.com/article/10.3390/ma18061207/s1, Table S1: Viscosity, *η*/mPa, of the base and formulated oils as a function of temperature at 0.1 MPa obtained with the Anton Paar Stabinger SVM 3000. Table S2: Density, *ρ*/g⋅cm^-3^, of base and formulated oils as a function of temperature at 0.1 MPa obtained with the Anton Paar Sta-binger SVM 3000.
