# Supplementary material for: Base Oils and Formulated Transmission Oils for Electrical Vehicles: Thermophysical and Tribological Properties
Source: Materials (Basel). 2025 Mar 8;18(6):1207. doi: 10.3390/ma18061207 (PMC11944221; doi:10.3390/ma18061207)
Supplement: Supplementary file 1 [file materials-18-01207-s001.zip › materials-3512868-supplementary.pdf]

Supplementary material

Base oils and formulated transmission oils for electrical vehicles: thermophysical and tribological properties

José M. Liñeira del Río [1,*], Alonso Alba [1], Martín Gómez Martínez [1], Alfredo Amigo [2], Josefa Fernández [1]

[1] Laboratory of Thermophysical and Tribological Properties, Nafomat Group, Department of Applied Physics, Faculty of Physics, Institute of Materials (iMATUS), Universidade de Santiago de Compostela, 15782, Santiago de Compostela, Spain
[2] Laboratory of Thermophysical and Surface Properties of Liquids, Department of Applied Physics, Faculty of Physics, University of Santiago de Compostela, 15782 Santiago de Compostela, Spain

* Correspondence: josemanuel.lineira@usc.es

Table S1

Viscosity, η/mPa·s, of the base and formulated oils as a function of temperature at 0.1 MPa obtained with the Anton Paar Stabinger SVM 3000.

T/K	η/mPa·s	T/K	η/mPa·s	T/K	η/mPa·s
		PAO6			
278.15	137.9	313.15	24.84	348.15	8.040
283.15	102.8	318.15	20.53	353.15	7.070
288.15	78.15	323.15	17.16	358.15	6.261
293.15	60.36	328.15	14.49	363.15	5.581
298.15	47.54	333.15	12.35	368.15	5.007
303.15	37.70	338.15	10.62	373.15	4.521
308.15	30.40	343.15	9.207		
		PAO8			
278.15	253.1	313.15	39.47	348.15	11.63
283.15	184.2	318.15	32.09	353.15	10.13
288.15	137.1	323.15	26.41	358.15	8.892
293.15	103.6	328.15	21.98	363.15	7.859
298.15	79.77	333.15	18.49	368.15	6.991
303.15	62.13	338.15	15.70	373.15	6.261
308.15	49.18	343.15	13.46		
		ATF CVT			
278.15	168.0	313.15	31.41	348.15	10.71
283.15	125.4	318.15	26.18	353.15	9.498
288.15	95.62	323.15	22.04	358.15	8.464
293.15	74.23	328.15	18.75	363.15	7.591
298.15	58.58	333.15	16.08	368.15	6.847
303.15	46.94	338.15	13.94	373.15	6.184

308.15	38.15	343.15	12.17		
		ATF DCT			
278.15	170.2	313.15	30.94	348.15	10.39
283.15	126.6	318.15	25.70	353.15	9.197
288.15	96.18	323.15	21.58	358.15	8.187
293.15	74.33	328.15	18.32	363.15	7.335
298.15	58.40	333.15	15.71	368.15	6.610
303.15	46.60	338.15	13.58	373.15	5.987
308.15	37.72	343.15	11.83		
		ATF VI			
278.15	146.8	313.15	25.81	348.15	8.537
283.15	108.5	318.15	21.37	353.15	7.543
288.15	81.91	323.15	17.90	358.15	6.704
293.15	62.96	328.15	15.16	363.15	6.002
298.15	49.45	333.15	12.96	368.15	5.402
303.15	39.14	338.15	11.19	373.15	4.892
308.15	31.57	343.15	9.739		
		G-III 3			
278.15	48.79	313.15	10.88	348.15	4.224
283.15	37.35	318.15	9.280	353.15	3.796
288.15	29.23	323.15	7.985	358.15	3.432
293.15	23.28	328.15	6.929	363.15	3.123
298.15	19.03	333.15	6.061	368.15	2.860
303.15	15.50	338.15	5.335	373.15	2.631
308.15	12.91	343.15	4.731		
		G-III 4			
278.15	83.43	313.15	16.29	348.15	5.774
283.15	62.70	318.15	13.66	353.15	5.136
288.15	48.09	323.15	11.58	358.15	4.600
293.15	37.54	328.15	9.912	363.15	4.147
298.15	30.02	333.15	8.558	368.15	3.762
303.15	24.05	338.15	7.450	373.15	3.433
308.15	19.66	343.15	6.535		
		G-III 6			
278.15	180.7	313.15	28.85	348.15	8.951
283.15	131.5	318.15	23.62	353.15	7.853
288.15	97.82	323.15	19.58	358.15	6.940
293.15	74.12	328.15	16.43	363.15	6.177
298.15	57.36	333.15	13.92	368.15	5.533
303.15	44.83	338.15	11.91	373.15	4.990
308.15	35.70	343.15	10.28		

Table S2

Density, ρ/g·cm^{-3}, of base and formulated oils as a function of temperature at 0.1 MPa obtained with the Anton Paar Stabinger SVM 3000.

T/K	ρ/g·cm^{-3}	T/K	ρ/g·cm^{-3}	T/K	ρ/g·cm^{-3}
			PAO6		
278.15	0.8329	313.15	0.8114	348.15	0.7897
283.15	0.8298	318.15	0.8084	353.15	0.7866
288.15	0.8268	323.15	0.8053	358.15	0.7834
293.15	0.8238	328.15	0.8021	363.15	0.7803
298.15	0.8207	333.15	0.799	368.15	0.7772
303.15	0.8176	338.15	0.7959	373.15	0.7740
308.15	0.8145	343.15	0.7928		
			PAO8		
278.15	0.8375	313.15	0.8163	348.15	0.7948
283.15	0.8345	318.15	0.8133	353.15	0.7918
288.15	0.8315	323.15	0.8102	358.15	0.7887
293.15	0.8285	328.15	0.8072	363.15	0.7856
298.15	0.8255	333.15	0.8041	368.15	0.7825
303.15	0.8224	338.15	0.801	373.15	0.7794
308.15	0.8194	343.15	0.7979		
			ATF CVT		
278.15	0.8544	313.15	0.8325	348.15	0.8103
283.15	0.8513	318.15	0.8294	353.15	0.8071
288.15	0.8482	323.15	0.8262	358.15	0.8039
293.15	0.8451	328.15	0.823	363.15	0.8007
298.15	0.842	333.15	0.8198	368.15	0.7975
303.15	0.8388	338.15	0.8167	373.15	0.7943
308.15	0.8357	343.15	0.8135		
			ATF DCT		
278.15	0.8259	313.15	0.837	348.15	0.8149
283.15	0.8228	318.15	0.8339	353.15	0.8117
288.15	0.8197	323.15	0.8307	358.15	0.8085
293.15	0.8166	328.15	0.8276	363.15	0.8053
298.15	0.8135	333.15	0.8244	368.15	0.802
303.15	0.8103	338.15	0.8212	373.15	0.7988
308.15	0.8072	343.15	0.8181		
			ATF VI		
278.15	0.8488	313.15	0.8271	348.15	0.8052

283.15	0.8457	318.15	0.824	353.15	0.8021
288.15	0.8426	323.15	0.8209	358.15	0.7989
293.15	0.8395	328.15	0.8178	363.15	0.7958
298.15	0.8364	333.15	0.8146	368.15	0.7926
303.15	0.8333	338.15	0.8115	373.15	0.7894
308.15	0.8302	343.15	0.8084		

G-III 3

278.15	0.8381	313.15	0.8159	348.15	0.7936
283.15	0.8349	318.15	0.8128	353.15	0.7903
288.15	0.8318	323.15	0.8096	358.15	0.7871
293.15	0.8286	328.15	0.8064	363.15	0.7838
298.15	0.8254	333.15	0.8032	368.15	0.7806
303.15	0.8223	338.15	0.8000	373.15	0.7774
308.15	0.8191	343.15	0.7968		

G-III 4

278.15	0.838	313.15	0.8161	348.15	0.7941
283.15	0.8349	318.15	0.813	353.15	0.7909
288.15	0.8318	323.15	0.8098	358.15	0.7877
293.15	0.8286	328.15	0.8067	363.15	0.7845
298.15	0.8255	333.15	0.8035	368.15	0.7814
303.15	0.8224	338.15	0.8004	373.15	0.7782
308.15	0.8193	343.15	0.7972		

G-III 6

278.15	0.8447	313.15	0.8234	348.15	0.8020
283.15	0.8417	318.15	0.8204	353.15	0.7989
288.15	0.8387	323.15	0.8173	358.15	0.7958
293.15	0.8356	328.15	0.8143	363.15	0.7927
298.15	0.8326	333.15	0.8112	368.15	0.7896
303.15	0.8295	338.15	0.8081	373.15	0.7865
308.15	0.8265	343.15	0.8050		
